# VOCAL COMPLAINT, AUDITORY-PERCEPTUAL ASSESSMENT OF VOICE AND VOCAL SELF-ASSESSMENT IN WOMEN WITH MORBID OBESITY

**DOI:** 10.1590/S0102-6720201500S100008

**Published:** 2015-12

**Authors:** Lourdes Bernadete Rocha de SOUZA, Leandro de Araujo PERNAMBUCO, Marquiony Marques dos SANTOS, Joana Cristina Vasconcelos da SILVA

**Affiliations:** Department of Speech Pathology of the Federal University of Rio Grande do Norte and Obesity and Related Diseases Surgical Center - SCODE, Natal, RN, Brazil

**Keywords:** Voice, Self-assessment, Morbid obesity, Bariatric surgery

## Abstract

***Background*:**

Obese people often have altered breathing patterns and therefore may experience difficulties in voice production.

**Aim:**

: To verify the presence of vocal complaints and the correlation between the auditory-perceptual analysis of voice and vocal self-assessment of a group of women with morbid obesity before and after bariatric surgery.

**Methods:**

: A longitudinal, exploratory, descriptive study of 21 morbidly obese women aged between 28 and 68 years, assessed before and after bariatric surgery, was performed. The women filled out a form containing identification data and type of vocal complaint. Perceptual evaluation of voice and vocal self-assessment were performed using a visual analog scale. For perceptual assessment of voice the women were asked to say three sentences from the Consensus Auditory-Perceptual Evaluation of Voice.

**Results:**

: Of the 21 patients, 14 (66.6%) reported vocal complaints, of which 10 (71%) vocal fatigue, eight (57.14%) voice failures and seven (50%) vocal effort. All participants reported improvements in the voice after surgery, irrespective of having reported vocal complaints before surgery. There was no correlation between vocal self-assessment and auditory-perceptual assessment of the voice before or after the procedure. There was no correlation between vocal self-assessment and perceptual evaluation of the voice before surgery.

**Conclusion:**

: Obesity interfered with voice production and influenced negative perception and therefore vocal complaints. Complaints about vocal production cannot be perceived by a speech therapist with the same impact as by patients, as both employ different criteria for vocal evaluation. Vocal self-assessment is an important tool in voice evaluation.

## Abstract (pt)

***Racional*:**

Pessoas obesas comumente possuem alteração do padrão respiratório e, desta forma, podem apresentar dificuldades na produção da voz.

**Objetivo:**

: Verificar a presença de queixa vocal e a correlação entre a análise perceptivo-auditiva e a autoavaliação da voz de um grupo de mulheres com obesidade mórbida antes e após a cirurgia bariátrica.

**Métodos:**

: Trata-se de estudo longitudinal, exploratório, transversal e descritivo. Participaram 21 mulheres com obesidade mórbida na faixa etária entre 28 e 68 anos, avaliadas antes e após procedimento bariátrico. Foi realizado o preenchimento de ficha contendo os dados de identificação e tipo de queixa vocal. Foi realizada a avaliação perceptiva e a autoavaliação da voz utilizando-se a escala analógica visual. Para análise perceptiva da voz foi solicitada emissão de três frases do Consenso da Avaliação Perceptivo Auditiva da Voz.

**Resultados:**

: Das 21 pacientes 14 (66,6%) apresentaram queixas vocais, sendo que 10 (71%) relataram cansaço ao falar, oito (57,14%) falhas na voz e sete (50%) esforço ao falar. Todas as participantes relataram melhora na voz após a operação, independente de não ter referido queixa vocal antes dela. Houve correlação entre a autoavaliação e a avaliação perceptiva antes e após o procedimento, e a autoavaliação e avaliação perceptiva da voz após ele. Não houve correlação entre a autoavaliação e a avaliação perceptiva da voz antes operação.

**Conclusão:**

: A obesidade interferiu na produção da voz e influenciou a percepção ruim e, por consequência, as queixas vocais. As queixas quanto à produção vocal, não podem ser percebidas pelo fonoaudiólogo com o mesmo impacto que é percebido pelos pacientes; ambos apresentam critérios distintos na avaliação vocal. A autoavaliação vocal mostrou ser ferramenta importante na avaliação vocal.

## INTRODUCTION

Obesity is a chronic condition caused by multiple factors. There are several treatment approaches, such as physical exercise, anti-obesity drugs and nutritional counseling. However, many patients do not respond to these measures, and require more drastic interventions, such as bariatric surgery, which has proved to be effective in the clinical management of cases of morbid obesity^1^ ^,2.^

Studies report that the abnormal deposition of adipose tissue in the upper airway can obstruct voice production^3^. Another factor that impacts vocal function as a result of obesity is reduced lung function, through an increase in the fatty tissue around the ribs and abdomen, which helps to reduce thoracic compliance and respiratory muscle strength in obese individuals^4^. As a result, it alters the physiology of the respiratory structures of the elements of phonation, resulting in difficulties in voice production in these individuals.

A recent study by Souza et al.^3^ aimed at verifying the fundamental frequency, maximum phonation time and vocal complaints of women with morbid obesity, found a high percentage of vocal complaints, mainly vocal fatigue and voice failure, complaints which were possibly related to the interference of respiratory function in voice production.

For the diagnosis of any vocal disease or disorder, evaluation of the voice throughout the entire treatment process is important, as it allows the evolution of the patient during speech therapy to be observed^5^. Vocal assessment requires multiple measures in order to describe all the features of vocal production, which is a complex phenomenon. Therefore assessment of vocal function and voice quality becomes more effective when defined by both the medical professional and patient^6^. 

In vocal assessment, it is important that the speech therapist always considers the patient's own perception of his or her own voice, as this is a relevant factor in prognosis. Vocal self-assessment is extremely useful and strives to identify the patient's perception of his or her own voice. As it is an assessment that is influenced by personality and by psychological factors, it is widely used to make comparisons with the objective measurements of the voice taken during an evaluation. It can be measured by questions, multiple choice options or the Visual Analogue Scale^7^. Therefore, the individual's perception of his or her own voice and the impact of this on quality of life complements the perception of health professionals of the degree of a disorder^8.^

The present study aimed to determine the presence of vocal complaints and the correlation between perceptual evaluation and self-assessment of the voices of a group of women with morbid obesity before and after bariatric surgery.

## METHODS

The present study was approved by the Human Research Ethics Committee of the institution, under reference number 207.630/2013.

A longitudinal, exploratory, cross-sectional, descriptive study was performed at the Obesity and Related Diseases Surgical Center (SCODE) of a university hospital. A total of 21 women with morbid obesity, evaluated before and after bariatric surgery, participated in this study. Data collection was performed from November 2012 to April 2014 and inclusion and exclusion criteria were applied. Patients with a body mass index above 35 kg/m^2^ were considered for inclusion while smokers and those with a virus or allergy at the time of vocal assessment were excluded.

The individuals signed a Letter of Free and Informed Consent to confirm their participation in the study. Subsequently, a brief interview was held to fill out an identification card with information about age, weight, voice complaints and type of complaint. 

Data collection was carried out by recording the voice using a laptop and the acoustic analysis software PRAAT version 5.3.16^9^. During collection, the patients sat with a microphone positioned 5 cm from their mouths. For perceptual evaluation of voice the individuals were asked to produce connected speech using three sentences from the Consensus Auditory-Perceptual Evaluation of Voice (CAPE-V)^10^ (Papai trouxe pipoca quente; Está na hora de acabar; Minha mãe namorou um anjo). Later, this recording was analyzed by a voice specialist with experience in auditory perceptual evaluation of voice quality. 

Both vocal self-assessment and perceptive evaluation of the voice of the population of this study was performed using the Visual Analogue Scale, a tool widely used in healthcare for the measurement of subjective phenomena. It consists of a 100 mm horizontal line, with excellent voice quality on the left and very poor voice quality on the right. To perform self-assessment, individuals were asked to mark a point on this line according how they classified their voice. A 10 cm ruler was then placed on the line to find the corresponding score of the point marked by the individual. The same procedure was carried out by the expert who evaluated the voices of participants.

Eight months after surgery, the patients were invited back to the center, completed the questionnaire on voice complaints, and again had their voices evaluated from auditory perceptual and self-assessment perspectives, using the same criteria as in the preoperative period.

Data was input into a database of the PSPP statistical software program. Quantitative variables were expressed according to measures of central tendency and dispersion. The nonparametric Wilcoxon test and Spearman's rank correlation coefficient were used for analysis. A statistical significance of 5% was considered. 

## RESULTS

The 21 women with morbid obesity were aged between 28 and 68 years with an average age of 41.33 (±11.26) years. Of these, 14 (66.6%) suffered from vocal complaints before the operation. Among the volunteers that reported vocal complaints, 10 described having vocal fatigue (71%), eight described voice failure (57.14%), seven vocal effort (50%) and six drying of the vocal tract (42.8%). After surgery none of the women reported vocal complaints.

Table 1 shows the descriptive analysis of the age range and weight of the individuals before and after surgery. It was found that the average weight loss of subjects was 33.56% of total weight prior to surgery.Table 2 shows that the results of self-assessment and perceptual evaluation by a speech therapist differed before and after surgery, with both measurements showing better results after surgery. Self-assessment revealed a higher average Visual Analogue Scale score, meaning that the patient's own perception of her voice was more negative than that of the speech therapist. Eight months after surgery, however, such differences no longer existed and the mean scores were very similar, indicating that after the operation both the patient and the speech therapist perceived the voice as being very similar. Table 3 shows that there was no correlation between self-assessment and perceptual evaluation by the speech therapist before and after surgery. 

**TABLE 1-:** Mean, standard deviation, minimum and maximum values by age range, weight before and after surgery

	**n**	**Minimum**	**Maximum**	**Mean and standard-deviation**
Age range	21	28	68	41.33 (± 11.26)
Weight				
Before surgery	21	80	137	115.76 (± 17.85)
After surgery	21	53	97	78.71 (± 12.24)
Weight loss	21	19	48	36.86 (± 8.446)

**TABLE 2:** - Median and Q.25 and Q.75 and p percentile values of self-assessment and auditory-perceptual assessment before and after surgery

	**Median**	**Q. 25**	**Q. 75**	**p**
Self-assessment				
Before	49,0	6,0	68,5	<0.001
After	5,0	2,5	20,0	
Auditory-perceptual assessment				
Before	22,0	11,5	30,5	0.001
After	10,0	6,0	12,0	

p<0.05 Wilcoxon test

**TABLE 3:** - Correlation between vocal self-assessment and auditory-perceptual evaluation of voice before and after surgery

	**rho**	**p**
Self-assessment before surgery x Auditory-perceptual assessment before surgery	0,411	0.064
Self-assessment after surgery x Auditory-perceptual assessment after surgery	0,338	0.134

p<0.05 Spearman's correlation test

## DISCUSSION

The aim of the present study was to verify the presence of vocal complaints and identify the correlation between self-assessment and auditory-perceptual assessment of the voice before and after bariatric surgery. It should be pointed out that both assessments are of great importance, as they complement and collaborate with each other in the treatment planning process.

All the participants of the study reported that their voice improved after bariatric surgery, regardless of whether they had reported vocal complaints or not before the operation, a result which agreed with the findings of other authors^11^ who argued that obesity can influence the components of voice production such as the expiration of air from the lungs, vocal fold vibration and the modification of sound through the resonant cavities, which may have led to most of the participants experiencing difficulties in voice production and reporting vocal complaints before surgery.

Preoperative vocal self-assessment by patients produced more negative results than did perceptual evaluation by a speech therapist. After bariatric surgery, the results of both vocal self-assessment and the perceptual evaluation of the speech therapist improved and the Visual Analogue Scales were more alike (Table 2). The more negative results before the operation may be explained by the fact that neck adiposity interferes with respiratory muscle strength^12^ and thus influences vocal production^3^. Obese individual may have vocal production difficulties ranging from pneumo-phono-articulatory incoordination, caused by the accumulation of fat in the abdominal wall, to changes in the vocal tract, also caused by the accumulation of fat^13^, factors that interfere with vocal production and consequently lead to vocal complaints. 

One study^11^ revealed that obesity interferes with the size and configuration of the aerodigestive tract, as well as affecting the volume of tissue in the vocal folds, which may lead to patients and speech therapists evaluating speech differently before and after weight loss. This clearly occurred in the present study where patients self-assessed their voices more negatively in the preoperative period than in the period after surgery.

There was no correlation between self-assessment and perceptual evaluation for either of the assessed time periods (Table 3). This lack of correlation may be explained by the fact that the patients does not judge voice quality solely in terms of listening, but also which they feel during voice production, including factors associated with physical sensations such as fatigue, stress and/or discomfort during vocal emission. The evaluation of the speech therapist is based more specifically on evaluation of the sound of the voice only. The professionals also has experience of a wider range of vocal disorders, and as a result, their reference points may be of voices with more serious disorders^9^ ^,14^. Therefore, it is possible to infer that the evaluations reflect different dimensions.

According to the results of the present study, it can be argued that before surgery, a reduction in lung compliance and increased chest wall tension, together with the decreased strength and endurance of the respiratory muscles^4^, may have hindered the ability to mobilize airflow and interfered with the coordination of breathing and speech, resulting in phonatory effort and hence the appearance of vocal complaints. It is noteworthy that the reduction of excess fat was of paramount importance in minimizing phonatory effort, a fact observed in both self-assessment and perceptual evaluation, including through the lack of vocal complaints after weight loss. 

The present study found that self-assessment strategies should be used in clinical practice to qualify and/or quantify vocal complaints, assisting the process of evaluation and treatment planning. Self-assessment is an indispensable complement to information gathered from perceptual evaluation, and can contribute to the targeting of intervention and treatment planning in clinical practice. 

## CONCLUSION

Obesity interfered with voice production and resulted in negative perception and therefore vocal complaints. Complaints about vocal production cannot be perceived by a speech therapist with the same impact as they are perceived by patients as such tests employ different vocal evaluation criteria. Vocal self-assessment proved to be an important tool in voice evaluation. 
